# Genome Sequence of a Novel Recombinant Coxsackievirus A6 Strain from Shanghai, China, 2013

**DOI:** 10.1128/genomeA.01347-14

**Published:** 2015-01-08

**Authors:** Xiaobo Feng, Wencai Guan, Yifeng Guo, Huiju Yu, Xiaoling Zhang, Ruhong Cheng, Zhen Wang, Zhen Zhang, Jia Zhang, Huaguo Li, Yin Zhuang, Hui Zhang, Zhiyong Lu, Ming Li, Hong Yu, Yixiao Bao, Yunwen Hu, Zhirong Yao

**Affiliations:** a Department of Dermatology, Xinhua Hospital, Shanghai Jiaotong University School of Medicine, Shanghai, China; b Pathogen Diagnosis and Biosafety Department, Shanghai Public Health Clinical Center, Fudan University, Shanghai, China; c Department of Pediatrics, Xinhua Hospital, Shanghai Jiaotong University School of Medicine, Shanghai, China; d Key Laboratory of Medical Molecular Virology, School of Basic Medical Science, Shanghai Medical College of Fudan University, Shanghai, China

## Abstract

A novel recombinant coxsackievirus A6 (CVA6) strain was isolated during a coxsackievirus A6 outbreak in Shanghai, China, in 2013. Genomic sequence and similarity plot analysis showed that the novel CVA6 strain shared higher similarity with a recent CVA4 strain rather than the recent CVA6 strain in the 2C and 3′ untranslated regions (UTRs).

## GENOME ANNOUNCEMENT

Coxsackievirus A6 (CVA6) belongs to the human enteroviruses species A (EV-A), genus *Enterovirus*, family *Picornaviridae*. Enterovirus infections resulted in a variety of diseases, such as hand-foot-month disease (HFMD). However, CVA6 gradually emerged and caused specially generalized exanthema in HFMD cases reported since 2008 (1–3). Although the prototype CVA6 strain isolated in 1949 has been available online since 2004, the recent CVA6 outbreak strain shizuoka18 isolated from Japan in 2011 was published in 2012 (4). CVA6 outbreaks have been detected in China since late 2012 (5–8), and a novel recombinant CVA6 strain was isolated and identified from the throat swabs of CVA6-positive specimens during this outbreak in Shanghai, China, in 2013.

To obtain the complete genome sequence of this novel CVA6 virus, viral RNA of a representative strain (PF1/SH/CHN/2013) was extracted from the culture of a rhabdomyosarcoma (RD) cell using a QIAamp viral RNA minikit (Qiagen, Santa Clara, CA). Overlapping fragments covering the viral genome were amplified using a one-step reverse transcription (RT)-PCR kit (TaKaRa), and the primers were designed mainly based on the recent CVA6 (GenBank accession no. AB678778) and CVA4 (GenBank accession no. HQ728260) genome sequences published in the previous studies (4, 9). The PCR products were purified and then sequenced on an automated sequencer ABI 3730 (Applied Biosystems, Foster City, CA). The sequenced DNA fragments were assembled into complete genomes using the ContigExpress project in Vector NTI version 11.5.

The whole-genome sequence of the novel recombinant CVA6 was 7,433 nucleotides (nt) long. A long single open reading frame was characterized. The polyprotein-coding sequence is 6,606 nt long and contains 2,201 codons. The 5′ and 3′ untranslated regions (UTRs) are 746- and 81-nt long, respectively. Compared with the recent CVA6 genome sequence (GenBank accession no. AB678778), a 1-nt deletion in the 5′ UTR and 697-nt differences with 51 amino acid changes in the coding region were found. Similarity plot analysis based on the whole genomes revealed that the 5′ UTR, P1 region, and partial P2 region (2A) were highly conserved between this novel CVA6 and recent CVA6 sequences, with at least 94.2% nucleotide sequence identity. However, in the 2C and 3′ UTR regions, the novel CVA6 strain shared high similarities of 91.0% to 93.5% with a recent CVA4 strain circulating in Shanghai. This study suggests the necessity of continuous monitoring of genetic recombination of more current CVA6 and other serotypes and provides a public health warning for possible outbreaks in the future.

### Nucleotide sequence accession number.

The genome sequence of the novel recombinant CVA6 strain has been deposited in GenBank under the accession no. KJ612513.
